# A Court Decision

**Published:** 1914-07

**Authors:** 


### A Court Decision.

A United States district court in the Southern District of Iowa has recently rendered a decision declaring vasectomy on wards of the State unconstitutional. Because of the growing interest in this question as a legislative measure, and its importance to society from a eugenistic point of view, the decision is produced below in full.

Every question has its different viewpoints, and in the name of justice it should be given due consideration. Especially is this true where the question involves the welfare of a nation and it is personal in its scope. But, in this particular question, it is difficult to believe, with the knowledge of the facts in the case so general, that there could be rendered a decision so garbled and distorted. It is painful to know that a court of justice should so grossly misrepresent the facts and the intent and purpose of such laws, and it is humiliating to see representative men, standing in the relation of dispensers of justice and truth, flaunt the old personal liberty "gag," so much resorted to by the cheap politician and demagogue.

As ground for the decision the court gives as its first count that the law is a "bill-of-attainder," in that it deprives the person concerned of a trial by jury and as controverting his individual rights to the pursuit of happiness, etc. In this the court overlooked the constitutional right of the State to protect the health and welfare of its citizens, a right it exercises (without trial) in the enforcement of health and quarantine laws. More imperative than the prevention of epidemics is the prevention of degenerates. Every child has the right (morally, biologically) to be born with a sound mind and body. If this right is not written in the Constitution, then the Constitution needs revision. The "personal liberty" of a man extends so far, only, as his acts do not interfere with the rights of others. That nation is freest where the individual makes the greatest personal sacrifices for the good of the whole.

On the second count the court holds that the law is unconstitutional because the punishment is "cruel and unusual." Here the court blindly refuses, or neglects, to understand the character of the punishment. From the reference to the barbaric ages, the common law and Blackstone's Commentaries, one would judge that castration (which is inhuman and harmful) is read into the act, whereas vasectomy is clearly stated as the procedure to be followed. Vasectomy is neither cruel nor unusual. It neither involves efficiency for, nor the right to, marital relations. It neither gives pain nor causes the loss of time. What it does is to prevent the transmission of harmful qualities that are inherent to unborn generations.

On the third count the court holds that the practice is humiliating, degrading and attended with mental suffering. Here again the mutilating operation of castration seems to have biased the judicial minds. To the normal man, the desire for offspring is natural, and to be without issue is humiliating in many instances. But any man imbued with the sense of responsibility to his child should accept with gratitude relief from this responsibility, especially as it does not entail the exercise of his personal prerogative. Therefore there is no cause for mental anguish or feeling of degradation.

There may be good reasons why such bills should not be enacted, but they are not on the grounds of unconstitutionality.

The writer believes there are better means than vasectomy to obtain the object intended. The intelligent regulation of procreation by preventive methods, not injurious to physiological functions, would benefit a vastly larger contingency of society. But for the positively defective classes, where high moral principles cannot be expected to direct the man's actions, vasectomy becomes an imperative duty of the State.

THE DECISION.

"The case," says the decision, "is one of diversity of citizenship, with Federal questions presented by a bill in equity with an application for a temporary injunction to restrain defendants as State officers from enforcing Chapter 187 of the Acts of the Thirty-fifth General Assembly (1913), authorizing a surgical operation called vasectomy on idiots, feeble-minded, drunkards, drug fiends, epileptics, moral and sexual perverts, and mandatory as to criminals who have been twice convicted of a felony. * * *

"Complainant in his verified bill alleges that the statute is in violation of the United States Constitution, in that it is in effect a bill of attainder in that there is to be no indictment or trial; that the statute abridges his privileges, and that he is denied the equal protection of the law; that he is denied due process of law; that the statute is in conflict with the Iowa Constitution in that the statute denies the inalienable right to enjoy life, liberty, and to pursue and obtain safety and happiness; that there is no jury trial awarded him, and that the statute provides cruel and unusual punishment.

"The case presents important questions. Statutes like this are of recent years, the first one upon the subject enacted less than fifteen years ago. The question has been before appellate courts but twice. In one case, that of State of Washington vs. Feilen, 126 Pac. Rep., 75 (L. R. A., N. S., 418), the statute was upheld. The court held that the punishment was not cruel or unusual in the constitutional sense. That case involved a most heinous offense, and the statute provided that in addition to life imprisonment jury and the court might determine whether he should be subjected to the operation of vasectomy. So that on the question now presented there is due process of law in that the matter was judicially determined. The other cases by the Supreme Court of New Jersey was that of Smith vs. Board of Examiners, 88 Atl. Rep., 963. In that case the operation was to be performed upon a woman who was an epileptic, an inmate of a State charitable institution, and that court held that the statute was based upon an unreasonable police regulation and denied to her and persons of her class the equal protection of the laws as guaranteed by the Fourteenth Amendment.

"In the twelfth century, Henry II declared it treason for any person to bring over any mandate from the Pope or anyone in authority in church affairs. This he made punishable as to secular clergymen by the loss of their eyes and by the operation.

"When Blackstone wrote his Commentaries he did not mention it as one of the cruel punishments, quite likely for the reason that with the advance of civilization the operation was looked upon as too cruel and was no longer performed. But each operation is to destroy the power of procreation. It is, of course, to follow the man during the balance of his life. The physical suffering may not be so great, but that is not the only test of cruel punishment; the humiliation, the degradation, the mental suffering are always present and known by all the public and will follow him wheresoever he may go. This belongs to the dark ages.

"As, of course, all persons concede that it would be better for society if some men did not beget children; diseased, deformed, mentally weak children and criminally inclined are brought into the world, oftentimes to their own shame and against the interest of the public. But are they not in the minimum? And must the marriage relation be formed under the newly-conceived laws based upon the brutalities of many centuries since and be allowed to take the place of the marriage relation formed along the true lines? Must the marriage relation be based and enforced by statute according to the teachings of the farmer in selecting his male animals to be mated with certain female animals only?

"It is somewhat difficult to define with precision what is cruel and unusual punishment in the constitutional sense. Usually the length of imprisonment following a conviction is within the discretion of the legislative body.

"No doubt delegates to the conventions, in providing against cruel punishment, had largely in mind what Blackstone had then recently written in Volume 4, page 376, such as being drawn or dragged to the place of execution, emboweling alive, cutting off the hands or ears, branding on the face or hand, slitting the nostrils, placing the prisoner in the pillory, the ducking, the rack and the torture, and, as in Spanish countries, crucifying. In a very few States of the Union the whipping post has been retained as a constitutional mode of punishment. But it will be found that the courts of those States have construed the statute thus imposing such punishment in the light of their history, and what had been done and was being done at the time of the adoption of their Constitution. No one can doubt but that under our present civilization if this surgical operation were to be adopted as a mode of punishment for any crime, all minds would so revolt that all courts without hesitation would declare it to be a cruel and unusual punishment. As we understand it, the operation was never inflicted after the revolution of 1688. So that if, as some now contend, it is now competent for a Legislature to impose such punishment as existed by the common law, the validity of the statute providing for it could not be upheld, because that punishment was one imposed back of the time of the common law, as, generally speaking, it comes down to us. In O'Neil vs. Vermont, 144 U. S., 323, and Weems vs. United States, 217 U. S., 349, and In re Kemler, 136 U. S., 436, all phases of the question are so presented as to leave nothing further to be said.

"And our conclusion is that the infliction of this penalty is in violation of the Constitution, which provides that cruel and unusual punishment shall not be inflicted.

"Under the habitual criminal laws of the State, if a prior conviction is relied on, the same must be pleaded and established by the evidence. But we have cases, this one included, in which the prior conviction has not been judicially established. But in Hayes vs. Missouri, 120 U. S., 68, it was said that due process of law and the equal protection of the laws was secured if the laws operated on all alike, and that all persons subject to the laws are treated alike under the limitations imposed. The cases are numerous and without conflict to such holdings, and further citation need not be made.

"But assuming that the prior convictions all appear of record, and assuming there is no conflict in the testimony and no difficulty in reaching the conclusion, but little or no advance is made in determining the question. If it be said that the statute automatically decides the question and nothing remains for the prison physician to do but to execute that which is already of record, then the statute becomes a bill of attainder. One of the rights of every man of sound mind is to enter into the marriage relation. Such is one of his civil rights, and deprivation or suspension of any civil right for past conduct is punishment for such conduct, and this fulfills the definition of a bill of attainder, because a bill of attainder is a legislative act which inflicts punishment without a jury trial, as is fully discussed and held in the case of Cummings vs. Missouri, 71 U. S., 277, the Federalist, No. 44, by Madison;

Justice Samuel F. Miller on the Constitution, 534; Watson on the Constitution, 733-738.

"We hold the statute to be void, and unite in holding that a temporary writ of injunction should be issued as prayed."

